# Increased Functional Connectivity of the Angular Gyrus During Imagined Music Performance

**DOI:** 10.3389/fnhum.2019.00092

**Published:** 2019-03-18

**Authors:** Shoji Tanaka, Eiji Kirino

**Affiliations:** ^1^Department of Information and Communication Sciences, Sophia University, Tokyo, Japan; ^2^Department of Psychiatry, School of Medicine, Juntendo University, Tokyo, Japan; ^3^Juntendo Shizuoka Hospital, Shizuoka, Japan

**Keywords:** emotion, episodic memory, fMRI, imagery, precuneus

## Abstract

The angular gyrus (AG) is a hub of several networks that are involved in various functions, including attention, self-processing, semantic information processing, emotion regulation, and mentalizing. Since these functions are required in music performance, it is likely that the AG plays a role in music performance. Considering that these functions emerge as network properties, this study analyzed the functional connectivity of the AG during the imagined music performance task and the resting condition. Our hypothesis was that the functional connectivity of the AG is modulated by imagined music performance. In the resting condition, the AG had connections with the medial prefrontal cortex (mPFC), posterior cingulate cortex (PCC), and precuneus as well as the superior and inferior frontal gyri and with the temporal cortex. Compared with the resting condition, imagined music performance increased the functional connectivity of the AG with the superior frontal gyrus (SFG), mPFC, precuneus, PCC, hippocampal/parahippocampal gyrus (H/PHG), and amygdala. The anterior cingulate cortex (ACC) and superior temporal gyrus (STG) were newly engaged or added to the AG network during the task. In contrast, the supplementary motor area (SMA), sensorimotor areas, and occipital regions, which were anti-correlated with the AG in the resting condition, were disengaged during the task. These results lead to the conclusion that the functional connectivity of the AG is modulated by imagined music performance, which suggests that the AG plays a role in imagined music performance.

## Introduction

The angular gyrus (AG; Brodmann area 39) resides in the posterior part of the inferior parietal lobule (IPL), which is greatly expanded in humans compared with other primates and is associated with higher cognitive functions (Fjell et al., 2015). The functions of the AG appear diverse, including attention, spatial cognition, conceptual representation, semantic processing, language, reasoning, social cognition, and episodic memory (Vincent et al., 2006; Binder et al., 2009; Seghier et al., 2010; Seghier and Price, 2012; Bonner et al., 2013; Seghier, 2013; Price et al., 2015). The AG is a rich-club node (Grayson et al., 2014), and its diverse functioning is substantiated by the widespread structural and functional connections with many brain regions (Seghier, 2013). Resting-state functional networks of the AG have been extracted by a functional connectivity analysis (Uddin et al., 2010), which is based on the correlation of blood-oxygen-level dependent (BOLD) time-series of a pair of brain regions or voxels. Previous studies further suggested that the AG is a connector hub (Xu et al., 2016) that is involved in several functional networks, including the default mode network (DMN) and the cingulo-opercular, fronto-parietal, and ventral attention networks (Igelström and Graziano, 2017). A review of the functions and networks of the AG suggested that “the AG emerges as a cross-modal hub where converging multisensory information is combined and integrated to comprehend and give sense to events, manipulate mental representations, solve familiar problems, and reorient attention to relevant information” (Seghier, 2013, p.43).

The association of the AG functions with music is worth studying for the following reasons. Music evokes emotions and influences moods (Koelsch, 2014). Using a novel sound-based theory-of-mind paradigm, a recent study (Bravo et al., 2017) suggested that the AG contributes to valence inferences to sound. Music performance is not just manipulation of a musical instrument but requires the control of various domains of information processing in performers, such as attention (Meltzer et al., 2015), working memory, musical semantics (Koelsch, 2011), emotion regulation, musical expressivity (Cespedes-Guevara and Eerola, 2018), and mentalizing (Downey et al., 2013; Cespedes-Guevara and Eerola, 2018). Because these functions emerge as network properties, it is intriguing to know how these networks are reconfigured during music performance. In an fMRI study, we recently analyzed the functional connectivity of the supplementary motor area (SMA) during imagined music performance as well as in the resting condition (Tanaka and Kirino, 2017). In the resting condition, the functional connectivity between the SMA and the AG was negative, which means the BOLD signals of these regions are negatively correlated or anticorrelated (Chen et al., 2011). This negative functional connectivity, indicating reciprocally inhibitory relationship between these regions, implies that these regions mediate functions in distinct domains (Jack et al., 2013). Given that the SMA is the center for motor planning (Carlsen et al., 2015; Hupfeld et al., 2017), this result suggests that the AG is not primarily involved in performance planning. It is plausible that the AG is involved in a network, which is distinct from the SMA network. In that case, a question arises as to what extent the functional connectivity of the AG is modulated by imagined music performance. Another study, using a musical imagery task, demonstrated the activation of the SMA, premotor cortex, intraparietal sulcus, and Wernicke’s area, as well as visual areas (Zhang et al., 2017). During the task, the participants imagined a music piece while watching a silent music visualization, which is a graphic music score. However, it is still unknown whether the AG is involved in performing the musical imagery task. Interestingly, a combination of mental imagery and music increased activation of brain regions associated with negative emotional and episodic memory processing, including the AG, as compared with music-only stimuli (Lee et al., 2016). Taken together with a suggestion that the AG plays a central role in pain reduction when fibromyalgia patients listen to music (Garza-Villarreal et al., 2015), it can be hypothesized that music is likely to influence the AG and its networks.

This study aimed to clarify whether the AG is involved in the processing of music performance. Our hypothesis was that the functional connectivity of the AG is modulated by imagined music performance. To test this hypothesis, we analyzed the functional connectivity of the AG during the imagined music performance task and the resting condition. If the task reconfigures the AG network, one could conclude that the AG plays a role in imagined music performance.

## Materials and Methods

### Ethics Statement

All study procedures were approved by the ethics committees of Sophia University and Juntendo University, Japan. This study also conformed to the tenets of the Declaration of Helsinki. All participants provided written informed consent prior to study participation.

### Participants

Participants who were graduate and undergraduate music school students were recruited by advertisement. Among right-handers, 41 were female and five were male. Because of fewer male participants, this study analyzed only the data from the 41 female participants (mean age: 23.4 years; age range: 19–30 years). All participants were Japanese, and healthy, without a history of neurological or neuropsychiatric disease. They had begun musical training at the age of 3–5 years (i.e., all participants had more than 15 years of musical training) and had actively participated in concert performances. These students specialized in classical music played on various instruments: 15 played the piano, eight the violin, four the clarinet, and 14 were opera vocalists.

### Tasks

All participants completed two sessions. The first was the imagined music performance session and the second was the resting-state session. Each session lasted 6 min and 40 s. During the imagined music performance session, the participants were asked to imagine the act of performing music in a concert hall as vividly as possible, with their eyes closed, and without performing actual movements. The “performed” music was chosen freely from their repertoires. For example, pianists chose a piece of piano music (e.g., Ballade No. 1 by Frederic Chopin); violinists chose a piece of violin music (e.g., Violin Sonata No. 1 by Robert Schumann); and vocalists chose an opera aria (e.g., “Regnava nel silenzio” from Lucia di Lammermoor by Donizetti). The performance was truncated at the end of each session. In the resting-state session, the participants were instructed to close their eyes and not think about anything in particular.

### Image Acquisition

#### Structural Images

Whole-brain images were acquired on a Philips Achieva 3.0-T magnetic resonance imaging (MRI) scanner equipped with a 32-channel head coil array. High-resolution T1-weighted images were collected for anatomical reference, using a 3D magnetization-prepared rapid acquisition gradient echo sequence: echo time (TE) = 3.3 ms, repetition time (TR) = 15 ms, flip angle = 10°, matrix size = 180 × 256 × 256, and voxel size = 1 × 1 × 1 mm^3^. The total image acquisition time was 3 min 31 s.

#### Functional Images

BOLD fMRI data were collected during the imagined music performance and the resting-state sessions. A T2*-weighted gradient-echo-planar imaging sequence was used with the following parameters: TE = 30 ms, TR = 2,000 ms, flip angle = 90°, field of view = 240 × 240 mm^2^, matrix size = 64 × 64, number of axial slices = 33, and voxel size = 3.75 × 3.75 × 4.00 mm^3^. Each session consisted of 200 scans. The image acquisition time was 6 min 40 s.

### Preprocessing

Imaging data were preprocessed using the CONN toolbox (Whitfield-Gabrieli and Nieto-Castanon, 2012), in conjunction with Statistical Parametric Mapping, version 12 (Wellcome Department of Cognitive Neurology, London, UK^1^), running on MATLAB, version R2016b (MathWorks, Inc., Natick, MA, USA). The individual fMRI data were co-registered to the T1 images. The first four volumes were discarded, and the remaining 196 volumes were subjected to preprocessing. The fMRI data were slice-timing corrected, realigned, and subsequently normalized to the standard Montreal Neurological Institute template, as implemented in the Statistical Parametric Mapping software platform. Image artifacts originating from head movement were processed using the ART-based scrubbing procedure as an artifact removal tool. Signal contributions from white matter, cerebrospinal fluid, and micro-head movements (six parameters) were regressed out from the data. Finally, the fMRI data were band-pass filtered (0.008–0.09 Hz) and functional images were smoothed spatially using a Gaussian filter kernel (full width at half-maximum = 8 mm) for the subsequent seed-to-voxel analysis.

### Statistical Analyses

#### Voxel-Level Analysis

A seed-to-voxel functional connectivity analysis was performed using the CONN toolbox. The seed was either the left or right AG. Pearson’s correlation coefficients were calculated between the time course of the left or right AG and the time courses of all other voxels in the gray matter, which provided a seed-to-voxel connectivity matrix. Positive and negative correlation coefficients defined positive and negative functional connectivity, respectively (Whitfield-Gabrieli and Nieto-Castanon, 2012). The correlation coefficients were then converted into normally distributed scores using Fisher’s transformation, and subsequently used in the population-level analysis. Within the same sample, the difference of functional connectivity between the two conditions, imagined music performance and resting, was statistically tested. A height threshold of *p* < 0.001 (uncorrected) was applied to individual voxels to define clusters. The significance level for the extracted clusters was then set to *p* < 0.05, with the family-wise error correction.

#### ROI Analysis

The region of interest (ROI)-to-ROI analysis of functional connectivity was also performed using the CONN toolbox. The set of ROIs implemented in the CONN toolbox was based on the Harvard-Oxford Atlas. For each subject, the residual BOLD time courses were extracted from 132 ROIs covering the whole brain, and the correlation coefficients were calculated from the time courses. The correlation coefficients were converted into normally distributed scores using Fisher’s transformation. The connectivity matrix of the AG with the remaining 130 ROIs was constructed. Within the same sample, the difference of functional connectivity between the two conditions, imagined music performance and resting, was statistically tested. The threshold for differences in the connectivity matrix between the two conditions (imagined music performance and resting) was set at *p* < 0.05, false discovery rate corrected.

## Results

**Figure 1:**
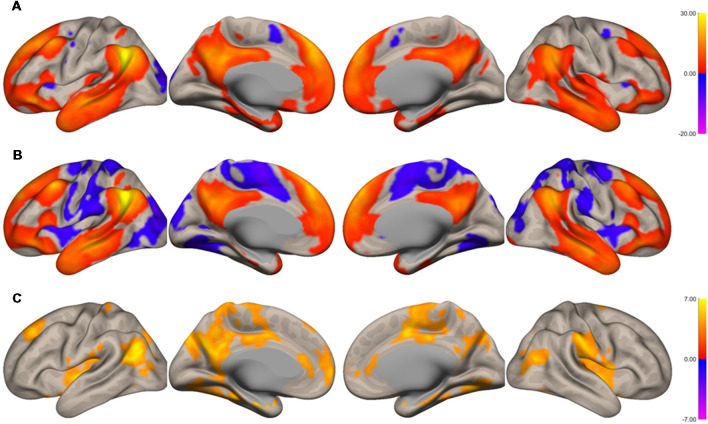
Surface maps of functional connectivity of the left angular gyrus (AG) as the seed region. **(A)** Imagined music performance. **(B)** Resting state. **(C)** Voxels with significant differences in connectivity with the left AG in imagined music performance vs. the resting condition. The significance level was peak-voxel *p* < 0.001, uncorrected, and cluster *p* < 0.05, family-wise error corrected.

**Figure 2:**
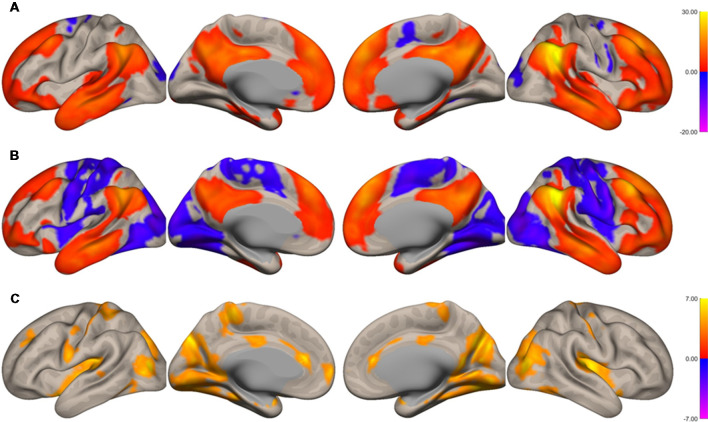
Surface maps of functional connectivity of the right angular gyrus (AG) as the seed region. **(A)** Imagined music performance. **(B)** Resting state. **(C)** Voxels with significant differences in connectivity with the right AG in imagined music performance vs. the resting condition. The significance level was peak-voxel *p* < 0.001, uncorrected, and cluster *p* < 0.05, family-wise error corrected.

**Figure 3:**
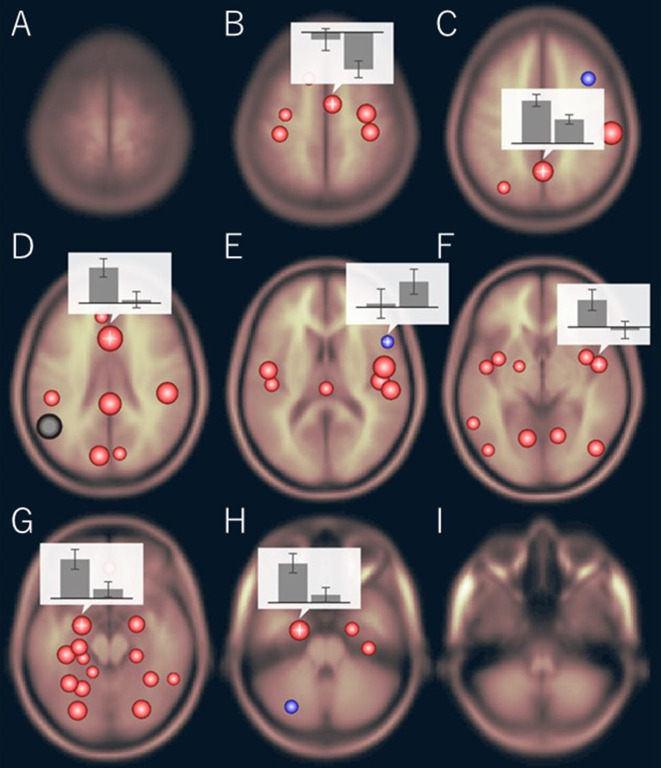
Region of interest (ROI)-to-ROI functional connectivity of the left AG as the seed during imagined music performance vs. resting condition. The bar graphs show the strength of the functional connectivity (left: performance, right: resting). **(B)** Right supplementary motor area (SMA), **(C)** precuneus, **(D)** anterior cingulate cortex (ACC), **(E)** right inferior frontal gyrus (IFG), **(F)** right planum polare, **(G)** left amygdala, **(H)** left anterior parahippocampal cortex. The significance level was *p* < 0.05, false discovery rate corrected.

The functional connectivity of the AG during the imagined music performance and the resting condition was analyzed. Figures 1, 2 show the seed-to-voxel functional connectivity maps of the AG as the seed. In the resting condition, the AG positively correlated with the superior frontal gyrus (SFG), inferior frontal gyrus (IFG), medial prefrontal cortex (mPFC), precuneus, posterior cingulate cortex (PCC), and temporal cortex. The connectivity of the AG with the SMA, sensorimotor, and occipital regions was negative, which means that the correlations of paired BOLD signals were negative or anti-correlated (Chen et al., 2011). Imagined music performance modulated the functional connectivity of the AG with many regions including the SFG, mPFC, cingulate cortex, precuneus, SMA, sensorimotor areas, operculum, superior temporal gyrus (STG), hippocampal/parahippocampal gyrus (H/PHG), amygdala, and occipital regions. Figure 3 shows the differences in ROI-to-ROI functional connectivity of the left AG as the seed between the imagined music performance and resting sessions. The functional connectivity of the right AG is not shown because the connectivity profiles were similar between the left and right AG.

There were three types of change in the functional connectivity of the AG during the imagined music performance. The first type (*Type 1*) is a further increase in functional connectivity from a positive value. The SFG, mPFC, precuneus, PCC, H/PHG, and amygdala were included in this type of increment. The second type (*Type 2*) is a change from insignificant connectivity to significantly positive connectivity. This type of increment included the anterior cingulate cortex (ACC) and STG. The third type (*Type 3*) is a change from significantly negative connectivity to insignificant connectivity. The SMA, sensorimotor, operculum, and occipital regions were included in this type of increment. Table 1 summarizes these three types of changes in the functional connectivity of the AG.

**Table 1:** Three types of change in the functional connectivity of the angular gyrus during imagined music performance compared with resting state.

	Target regions	Type of change
Type 1	SFG, mPFC, precuneus, PCC, H/PHG, amygdala	Enhancement
Type 2	ACC and STG	Engagement
Type 3	SMA, sensorimotor cortex, operculum/insula, and occipital regions	Disengagement

*The differences in functional connectivity between the angular gyrus (AG) and target regions listed in this table were statistically significant (*p* < 0.05, family-wise error corrected). ACC, anterior cingulate cortex; H/PHG, hippocampal/parahippocampal gyrus; PCC, posterior cingulate cortex; SFG, superior frontal gyrus; SMA, supplementary motor area; STG, superior temporal gyrus*.

## Discussion

### Types of Connectivity Change

In this study, we analyzed the functional connectivity of the AG during the imagined music performance task vs. the resting condition. The results show that the task induced selective changes in the functional connectivity of the AG with many brain regions. The lateralization of the reconfiguration profile was modest. We classified these changes into three clusters according the types of changes of the functional connectivity (Table 1). In *Type 1*, the connectivity of the AG with the SFG, mPFC, precuneus, PCC, H/PHG, and amygdala was significantly positive at rest and was further increased to be higher significantly positive during the task. In *Type 2*, the connectivity was insignificant at rest. The AG engaged the ACC and STG by increasing its connectivity with these regions to be significantly positive during the task. *Type 3* is the case that the connectivity was significantly negative at rest and became insignificant during the task. The target regions included the SMA, sensorimotor cortex, operculum, and occipital regions. We interpret these three types of change as follows.

### Enhancement of the Connectivity With the SFG, mPFC, Precuneus/PCC, H/PHG, and Amygdala

The connectivity of the AG with the SFG, mPFC, precuneus/PCC, H/PHG, and amygdala was enhanced during imagined music performance. The SFG is a principal hub connecting many brain regions (Ottet et al., 2013). The AG and SFG, as well as the mPFC and precuneus, are activated in social cognitive emotion tasks (Xie et al., 2016; Schmälzle et al., 2017). The internal representation of a desired personal goal activates the SFG, precuneus, and AG (Strauman et al., 2013). The SFG has also been associated with cognitive reappraisal of emotions (Falquez et al., 2014; Moore et al., 2016), introspection (Goldberg et al., 2006), and metacognition of self-relevance (Schmitz et al., 2004). The mPFC has been associated with self-processing (D’Argembeau et al., 2007; Benoit et al., 2010). The mPFC and precuneus/PCC are reliably activated by self-projection, episodic memory, and prospection (Buckner and Carroll, 2007). The precuneus and PCC are involved in the manipulation of mental imagery (Cavanna and Trimble, 2006), and these regions are involved in the processing of autobiographical memory (Spreng and Grady, 2010; Addis et al., 2012). In particular, these brain regions play a pivotal role in the construction of auditory scene, the mental representation of sounds (Spada et al., 2014). Consistent with this, a recent functional connectivity analysis showed enhanced functional networks of the precuneus in musicians relative to non-musicians (Tanaka and Kirino, 2016). Mental imagery facilitates music performance and is important for artistic proficiency (Keller, 2012). The SFG, mPFC, precuneus/PCC, and H/PHG constitute the DMN (Raichle and Snyder, 2007; Andrews-Hanna, 2012). Our results suggest that the DMN is actively involved in imagined music performance. Thus, our results support the revised view that the DMN is not task-negative but involved in active tasks (Spreng, 2012; Sormaz et al., 2018). Such tasks are internally focused or inherently generative, as is the imagined music performance task in this study.

The functional connectivity of the AG with the H/PHG and amygdala was also enhanced during the imagined music performance compared with the resting condition. The H/PHG and amygdala constitute the medial temporal limbic system and play central roles in the processing of emotions and memory (Frühholz et al., 2014). Auditory stimuli, such as vocal expressions and musically expressed emotions, elicit activation in the amygdala (Frühholz et al., 2014). The hippocampal regions are associated with episodic memory processing (Allan et al., 2000; Rugg and Vilberg, 2013). Both regions become active when listening to music (Koelsch et al., 2006; Trost et al., 2012; Frühholz et al., 2014). This result is consistent with previous studies suggesting that the H/PHG mediates memory-aided constructive simulation (Andrews-Hanna, 2012), which is required in imagined music performance. It might also be relevant to the recent fMRI study reporting that the functional connectivity between the AG and the parahippocampal cortex was increased during a sound-based theory-of-mind task, in which participants categorized stimuli of different sensory dissonance level in terms of positive/negative valence (Bravo et al., 2017).

### Engagement of the ACC and STG

The ACC and STG were engaged during imagined music performance. The ACC has been suggested to mediate emotional processing and appraisal (Etkin et al., 2011, 2015) as well as affective evaluation of performance monitoring (Braem et al., 2017). The STG includes the auditory areas. The primary auditory cortex, located in Heschl’s gyrus (HG), processes basic features of sound (Warrier et al., 2009; Brewer and Barton, 2016). The processing of sound pitch is mediated by HG and the planum temporale (PT; Hall and Plack, 2009). Sounds that vary in pitch to produce a melody activate HG, the PT, and the planum polare. The increased connectivity of the AG with the ACC and STG might enable the association between sound and emotion. The engagement of these regions, therefore, suggests the involvement of the AG in the emotional processing of “imagined” auditory information during the task.

### Disengagement of the SMA, Sensorimotor Cortex, Operculum/Insula, and Occipital Regions

The functional connectivity of the AG with the SMA, sensorimotor cortex, operculum, and occipital regions was negative at rest and was very weak during the imagined music performance. Previous research has demonstrated that the functional connectivity of the SMA with cortical areas, including the PFC, superior parietal lobule, and temporal cortical areas, increases during imagined music performance, possibly reflecting the processing for performance planning (Tanaka and Kirino, 2017). Disengagement of the SMA, sensorimotor cortex, operculum/insula, and occipital regions during imagined music performance, therefore, suggests that the AG network is involved in distinct aspects of imagined music performance.

Though not clearly shown in the result of the seed-to-voxel analysis, the ROI-to-ROI analysis showed that the functional connectivity between the AG and the IFG was decreased from significantly positive to insignificant. That is, the IFG was disengaged from the AG network. Since the IFG has been associated with syntax processing in music as well as in language (Maess et al., 2001; Kunert et al., 2015; Cheung et al., 2018), this result suggests that the AG is not involved in musical syntax processing.

### Limitations of This Study

The enhanced connectivity of the AG with regions such as the SFG, mPFC, precuneus, PCC, H/PHG, and amygdala indicates that imagined music performance requires various kinds of information processing, including introspection, mentalizing, scene construction, emotion regulation, social cognition, and aesthetic experience. Although this result is novel, this study, using a naturalistic task, could not narrow down the functions to those critically involved in imagined music performance. To do this, an appropriately designed control task would also be needed.

Because of fewer male participants, this study analyzed the data from female participants. There could be sex difference in the functional connectivity of the AG. This issue also should be addressed in the future.

## Conclusion

This study analyzed the functional connectivity of the AG and found increased connectivity with the SFG, mPFC, precuneus/PCC, H/PHG, and amygdala during the imagined music performance task. The connectivity with the ACC and STG was newly engaged or added to the AG network during the task. In contrast, the SMA, sensorimotor cortex, operculum, and occipital regions, which were anti-correlated with the AG during rest, were disengaged during the task. These results (i.e., the selective modulation of the functional connectivity of the AG with several brain regions) lead to the conclusion that the functional connectivity of the AG is modulated by imagined music performance, which suggests that the AG plays a role in imagined music performance.

## Author Contributions

ST and EK planned and conducted all the experiments. ST analyzed the data and wrote the manuscript.

## Conflict of Interest Statement

The authors declare that the research was conducted in the absence of any commercial or financial relationships that could be construed as a potential conflict of interest.

## Footnotes

^1^http://www.fil.ion.ucl.ac.uk/spm
