# Corrigendum: Increased Functional Connectivity of the Angular Gyrus During Imagined Music Performance

**DOI:** 10.3389/fnhum.2021.716376

**Published:** 2021-07-07

**Authors:** Shoji Tanaka, Eiji Kirino

**Affiliations:** ^1^Department of Information and Communication Sciences, Sophia University, Tokyo, Japan; ^2^Department of Psychiatry, School of Medicine, Juntendo University, Tokyo, Japan; ^3^Juntendo Shizuoka Hospital, Shizuoka, Japan

**Keywords:** emotion, episodic memory, fMRI, imagery, precuneus

In the original article, there were errors. The authors made typographical errors.

Page 3, Statistical Analyses/Voxel-Level Analysis:

In the third and fourth lines, both SMA should be replaced by AG.

The authors apologize for these errors and state that these do not change the scientific conclusions of the article in any way. The original article has been updated.

